# Chromatin Remodeling and Epigenetic Regulation in Plant DNA Damage Repair

**DOI:** 10.3390/ijms20174093

**Published:** 2019-08-22

**Authors:** Jin-Hong Kim

**Affiliations:** Advanced Radiation Technology Institute, Korea Atomic Energy Research Institute, 29 Geumgu-gil, Jeongeup-si, Jeollabuk-do 56212, Korea; jhongkim@kaeri.re.kr; Tel.: +82-63-570-3333

**Keywords:** chromatin, chromatin remodelers, histone modifiers, DNA (de-)methylation enzymes, genome stability, DNA repair, DDR signaling

## Abstract

DNA damage response (DDR) in eukaryotic cells is initiated in the chromatin context. DNA damage and repair depend on or have influence on the chromatin dynamics associated with genome stability. Epigenetic modifiers, such as chromatin remodelers, histone modifiers, DNA (de-)methylation enzymes, and noncoding RNAs regulate DDR signaling and DNA repair by affecting chromatin dynamics. In recent years, significant progress has been made in the understanding of plant DDR and DNA repair. SUPPRESSOR OF GAMMA RESPONSE1, RETINOBLASTOMA RELATED1 (RBR1)/E2FA, and NAC103 have been proven to be key players in the mediation of DDR signaling in plants, while plant-specific chromatin remodelers, such as DECREASED DNA METHYLATION1, contribute to chromatin dynamics for DNA repair. There is accumulating evidence that plant epigenetic modifiers are involved in DDR and DNA repair. In this review, I examine how DDR and DNA repair machineries are concertedly regulated in *Arabidopsis thaliana* by a variety of epigenetic modifiers directing chromatin remodeling and epigenetic modification. This review will aid in updating our knowledge on DDR and DNA repair in plants.

## 1. Introduction

**Figure 1:**
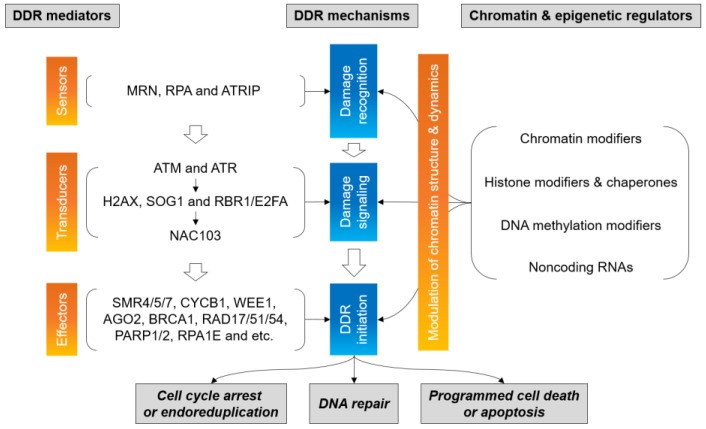
Signaling pathway of DNA damage response (DDR) in the context of chromatin. Chromatin structure and dynamics are regulated by chromatin remodeling and epigenetic modifications to mediate DNA damage recognition, signaling, and repair.

Plants are continually exposed to endogenous cellular processes and exogenous environmental events, which can compromise genome integrity through DNA damage. To counteract the detrimental effects of these processes or events, cells have developed a major and evolutionarily conserved defense mechanism known as DNA damage response (DDR). The eukaryotic DDR constitutes a complicated signaling pathway to sense or suppress DNA damage and facilitate DNA repair in the context of chromatin (Figure 1). Since chromatin mobility contributes to and equally endangers genome stability [1], understanding chromatin dynamics is important in regulating DDR in eukaryotes [2]. Both ATAXIA TELANGIECTASIA MUTATED (ATM) and ATM and RAD3-RELATED (ATR), initial DNA damage signaling kinases, are activated by chromatin perturbations for the induction of DDR, such as DNA repair, cell cycle checkpoints, cell death, and senescence [3]. In addition, concerted chromatin modifications orchestrate the repair process of DNA double-strand breaks (DSBs) by influencing the access and kinetics of the repair machinery and the choice of repair factors [4,5]. Chromatin composition and regulation is also associated with the relocation of repair sites to the nuclear periphery for homologous recombination (HR) repair of DSBs in heterochromatin [6].

During DDR, the proteasome-mediated histone loss from yeast chromatin is induced by the DNA damage checkpoint and the INOSITOL-REQUIRING MUTANT80 (INO80) chromatin remodeler, and this results in enhanced chromatin mobility and HR repair [7]. Chromatin remodelers and histone chaperones carry out the architectural tasks for chromatin and nucleosome dynamics in DNA damage and repair [8]. Therefore, chromatin regulators, such as chromatin remodelers and histone modifying enzymes are associated with genome stability as potential gatekeepers and signaling coordinators for the maintenance of genome integrity [9]. For example, the sequential acetylation and ubiquitination of histone H2A variant H2AX by the TIP60–UBC13 complex regulates the release of H2AX from damaged chromatin and enhances chromatin dynamics [10]. The INO80 chromatin remodeler binds phosphorylated H2AX (γH2AX) at DSB sites and regulates the abundance and positioning of nucleosomes for proper execution of DNA repair [11]. In addition, there is accumulating evidence that other epigenetic modifiers, such as DNA (de-)methylation enzymes and noncoding RNAs modulate epigenetic codes of the chromatin structure and affect genome stability and DNA repair [12,13,14].

Chromatin dynamics is necessary for cell cycle progression, which is finely coordinated by developmental and environmental signals in plants [15]. The chromatin structure is crucial for genome replication, transcriptional silencing, and DNA repair and recombination in plants [16]. It is also regulated for proper transcriptional induction in plants. In *Arabidopsis*, histone H2A variant H2AZ is enriched within the gene bodies of transcriptionally variable genes, while trimethylation of histone H3 lysine 4 (H3K4me3) is associated with transcriptional activation of stress responsive genes [17]. Concerted flexibility of chromatin structure and epigenetic marks influence genome accessibility and function in plant stress responses [18]. Although previous reviews have analyzed DNA damage signaling and repair in plants [19,20,21,22], there is need to update the available information on plant DDR and DNA repair in terms of chromatin remodeling and epigenetic regulation.

## 2. DNA Damage Signaling in the Context of Chromatin

The DDR signaling pathway is orchestrated by the master signaling kinases, ATM and ATR (Figure 1). The sensor proteins of this pathway recognize DNA and chromatin structures induced by DNA damage, while the transducers such as ATM, ATR, and their downstream kinases activate the effector proteins in a broad range of cellular processes for the maintenance of genome stability [22,23]. Nucleosome recognition by DDR sensors and transducers initiates or mediates DNA damage signaling and repair within the chromatin [24]. The MEIOTIC RECOMBINATION11 (MRE11)/RAD50/NBS1 (MRN) complex, a DDR sensor, recognizes DNA ends and chromatin at DSB sites, while the ATM bound to the MRN is activated by DNA ends and works as an initial signal transducer [25]. In contrast, ATR is only activated when ssDNA and ssDNA/dsDNA junctions at DNA damage sites are recognized by DDR sensors, such as REPLICATION PROTEIN A (RPA) and ATRIP [26,27]. The active forms of ATM/ATR kinases phosphorylate and/or regulate the secondary downstream transducers H2AX, SUPPRESSOR OF GAMMA RESPONSE1 (SOG1), and RETINOBLASTOMA RELATED1 (RBR1)/E2FA [22,28,29]. The phosphorylated SOG1 plays a pivotal role in plant DDR by inducing transcription of the tertiary downstream transducer NAC103 and the effectors SMR4/5/7, CYCB1, WEE1, ARGONAUTE2 (AGO2), BREAST CANCER1 (BRCA1), RAD17/51/54, PARP1/2, and RPA1E [30,31,32].

Nucleosomes and higher order chromatin structures are rearranged by chromatin remodeling during DDR. Chromatin remodeling at DNA damage sites as well as the binding of specific chromatin proteins, such as γH2AX and H2AZ to damaged DNA can affect the damage recognition, signaling, and repair processes [33,34]. Accumulating evidence shows how chromatin remodelers modulate DNA damage signaling and repair in eukaryotes. The human NuRD chromatin-remodeling complex accumulates within DSB-flanking chromatin and orchestrates proper signaling and repair of DSBs by promoting histone ubiquitylation at DSB sites to facilitate the accumulation of BRCA1 and the E3 ubiquitin ligase RNF168 [35]. The chromatin remodeler SMARCA5/SNF2H interacts with RNF168 in a DNA damage- and PARP-dependent manner and is required for the RNF168-dependent signaling of DSBs to stimulate γH2AX ubiquitylation and BRCA1 accumulation at DSB sites [36]. The yeast INO80 complex binds γH2AX at DSB sites and influences the dynamics of both γH2AX- and H2AZ-containing nucleosomes around DSBs for signaling and repair [11]. In plants, ATP-dependent chromatin remodelers (ACRs) or chromatin remodeling ATPases are divided into six major subfamilies, namely, INO80, SWITCH2/SUCROSE NONFERMENTABLE2 (SWI2/SNF2)-RELATED1 (SWR1), CHROMODOMAIN HELICASE DNA1 (CHD1), IMITATION SWITCH (ISWI), RADIATION-SENSITIVE54 (RAD54), and SNF2 [21,37]. Among them, the INO80, SWR1, and RAD54 chromatin remodeling complexes have proved to play an important role in plant DDR. The specific roles of plant chromatin remodelers in DNA damage signaling and repair will be discussed in Section 5.1.

## 3. DNA Damage Repair in the Context of Chromatin

Chromatin and nucleosome dynamics in both unicellular and multicellular eukaryotes are important in DNA repair as well as DNA damage sensing and signaling [8,24]. Many features of chromatin remodeling and DNA repair are commonly found in fungi, animals, and plant, despite distinct differences in detail [21,22]. Repairing DSBs and genome stability requires extensive chromatin remodeling to promote the formation of relaxed chromatin structures for the access of DNA-repair machinery at DSB sites in fungi, animals, and plants [20,38,39]. Other DNA damages such as single-strand gaps, nicks, stalled forks, alternative DNA structures, and base lesions must also be repaired in the chromatin context with similarities and differences to DSB repair [40]. Moreover, plant heterochromatin undergoes large-scale remodeling to repair DNA damage by creating a compartment with low chromatin density [41]. In this regard, updating our knowledge of DNA repair mechanisms in the context of chromatin is crucial for the engineering of plant genomes via both traditional and targeted mutagenesis [42].

The major DNA repair mechanisms such as HR, non-homologous end-joining (NHEJ), base excision repair (BER), nucleotide-excision repair (NER), and mismatch repair (MMR) are subject to a variety of chromatin remodeling in eukaryotic cells as follows (Table 1) [43].

HR: The *Arabidopsis* chromatin remodelers of the SWI2/SNF2 family involved in DDR and HR [61]. The *Arabidopsis* RAD54 and the DECREASED DNA METHYLATION1 (DDM1), which have the characteristic ATPase/helicase motifs of the SWI2/SNF2 family proteins, were found to contribute to DDR and HR repair after γ-irradiation by inducing chromatin structural changes through interaction with AtRAD51 [39,44]. In contrast, the human E3 ubiquitin ligase RNF20 modulates the chromatin structure for the initiation of HR repair by the ubiquitylating histone H2B through interaction with the histone chaperone FACILITATES CHROMATIN TRANSCRIPTION (FACT) [47]. Similarly, the *Arabidopsis* MMS21, a small ubiquitin-related modifier E3 ligase, functions in DDR and HR repair as a critical subunit of the STRUCTURAL MAINTENANCE OF CHROMOSOMES5/6 (SMC5/6) complex [45,46,62]. The *Arabidopsis* NSE4 subunit of the SMC5/6 complex may be involved in repair of zebularine-induced DNA-protein crosslinks [63]. 

NHEJ: The yeast chromatin remodeler RSC complex is recruited to DSB sites and facilitates NHEJ repair of DSBs depending on the presence of MRE11 and KU70/80 proteins [49]. The NHEJ repair in eukaryotic cells requires the ATM- and INO80-dependent nucleosome disassembly around DSBs [21,43] and is followed by the histone chaperones ASF1A, HIRA, and CHROMATIN ASSEMBLY FACTOR 1 (CAF-1)-dependent nucleosome reassembly in mammalian cells [48].

NER: The ACRs, which are conserved from the unicellular yeasts to the multicellular plants and humans, play a regulatory role in NER by reorganizing the chromatin structure and controlling DNA accessibility [50]. The SWITCH/SUCROSE NONFERMENTABLE (SWI/SNF) and INO80 complexes promote the removal of UV-induced DNA lesions and restore the chromatin structure by ATP-dependent chromatin remodeling during and after NER [51,52,53].

BER: The SWI/SNF-induced chromatin remodeling is also required for BER of 8-oxo-7,8-dihydroguanine (8-oxoG), the major reactive oxygen species-induced oxidative lesion in conventional nucleosomes, by perturbing the histone-DNA interactions to facilitate transcription as well as DNA repair [57,58]. The utility of reconstituted BER and chromatin systems indicates that interdependent cellular processes such as post-translational histone modification and ATP-dependent chromatin remodeling affect the accessibility of BER enzymes to nucleosomal DNA [54,55,56].

MMR: The proliferating cell nuclear antigen PCNA interacts with the MSH6 subunit of the mismatch recognition factor MutSα (MSH2-MSH6) as well as with CAF-1 and governs the balance between MMR and chromatin assembly in human cells [60]. Chromatin remodeling and histone modifications regulate MMR in eukaryotic cells by affecting nucleosome assembly and disassembly [59]. The specific roles of plant chromatin remodelers in DNA damage signaling and repair are detailed in Section 5.1. 

## 4. Epigenetic Regulation for DNA Damage Signaling and Repair

**Figure 2:**
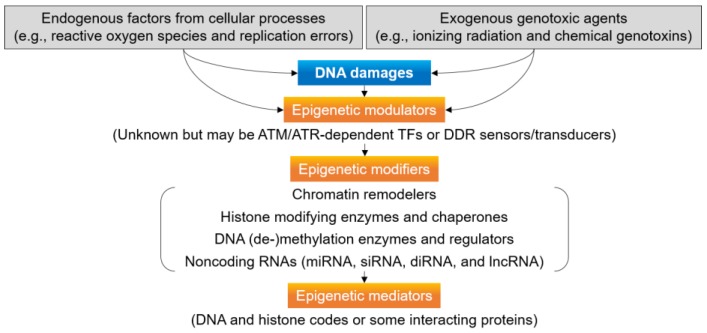
Epigenetic regulation of plant DDR. Epigenome integrity challenged by DNA damages mediates DDR but is restored by resetting the epigenomes structures via chromatin remodeling, histone modification, DNA methylation modification, and RNA-assisted silencing.

In DDR, the genome and epigenome integrity is coordinately maintained [64]. Epigenome integrity is challenged by DNA damages and it mediates DDR; however, it can be restored by resetting the epigenome structures to end DDR (Figure 2). In addition to chromatin remodeling and dynamics, other epigenetic changes, such as DNA methylation, histone modification, and RNA-assisted silencing, have been identified in genome maintenance, as well as DNA damage signaling and repair pathways [22]. For example, the ionizing radiation (IR)-induced genome instability, bystander, and transgenerational effects are suggested to be epigenetically regulated [65]. Histone modifications such as phosphorylation and ubiquitylation of H2AX are necessary for the recognition and signaling of a DSB and opening of chromatin to repair the lesion [34]. Expression of DNA repair genes is associated with DNA replication machinery-dependent histone modifications [66]. The oxidatively modified DNA base 8-oxoG in G-quadruplex-forming sequences can serve as an epigenetic sensor and signaling agent for gene activation by guiding BER in a gene promoter [67]. Epigenetic modifications are also involved in the environmental stress-induced genome rearrangement and may be inherited as an epigenetic stress memory to cope with subsequent stress [68,69].

The proposed framework for epigenetic regulation includes three types of genes encoding epigenetic modulators, modifiers, and mediators [70]. Epigenetic modulators activate or repress the epigenetic machinery, while the epigenetic modifiers modify DNA methylation or the chromatin structure, and epigenetic mediators are regulated by epigenetic modifiers (Figure 2). In DDR, epigenetic modifiers mediate DNA damage signaling and repair by changing epigenome integrity via chromatin remodeling, histone modification, DNA (de-)methylation, and RNA silencing. The ATM/ATR kinases and RNF8/RNF168 ubiquitin ligases help to recognize a DSB and open chromatin for repair by phosphorylating and ubiquitylating H2AX, respectively [23,71,72]. The H3K36 trimethyltransferase SETD2 is involved in the generation of an epigenetic histone maker H3K36me3 to recruit the recognition factor MutSα for MMR in human cells [73]. The H3K36me3-mediated MMR protects actively transcribed genes against mutation, not only during replication, but also during transcription [74]. In addition, non-coding RNAs directly affect the chromatin structure, transcription, and splicing, as well as facilitate DDR signaling and DNA repair via sequence-specific chromatin modification [75].

The epigenetic modifiers in plants, which have been identified to be involved in epigenetic regulation, can be divided into five groups according to their functions as follows: Regulators of DNA modification, histone-modifying enzymes and histone variants, polycomb proteins and interacting components, nucleosome-organizing proteins, and RNA-mediated gene silencing components [76]. For example, the *Arabidopsis* DNA methyltransferase CHROMOMETHYLASE3 (CMT3) is associated with non-CG DNA methylation and transcription of the inactive repeat sequences such as *CEN* and *TSI* after gamma irradiation [77]. Histone trimethyltransferases and/or acetyltransferases may induce histone modifications associated with the transcription of some DDR genes in *Arabidopsis* after γ-irradiation [78]. In addition, small noncoding microRNAs (miRNAs) in plant and animal genomes are responsive to IR-induced oxidative stress and may be responsible for the epigenetic regulation of some DDR genes [79,80]. The specific roles of plant epigenetic modifiers in DNA damage signaling and repair are detailed in Section 5.2, Section 5.3 and Section 5.4.

## 5. Chromatin and Epigenetic Modifiers for DDR

Eukaryotic cellular machineries for DNA replication, transcription, and repair need to maintain the proper spatial and temporal epigenetic codes in the genome [64,81]. Histone and DNA modifications as well as ACR-mediated chromatin remodeling function coordinately in epigenetic regulation to facilitate the effectiveness of the DDR [43,82]. Therefore, the specific roles of epigenetic regulators or modifiers, which have been identified in plants, are discussed in connection with DDR in four major groups as follows: Chromatin remodelers, histone modifiers, DNA (de-)methylation enzymes, and non-coding RNAs (ncRNAs; Table 2) [76].

### 5.1. Chromatin Remodelers

In yeasts, chromatin remodeling by the RSC complex is required for NHEJ repair of chromosomal DSBs [49]. The human NuRD complex is also involved in the signaling and repair of DSBs [35]. Moreover, plant genome stability is regulated in the context of DSB repair and chromatin structure maintenance [20,21]. Although various types of ACRs implicated in DDR have been identified in yeasts and mammals [38,43,83], there is a lack of experimental data to support the roles of plant chromatin remodelers in DDR [21,61]. However, some of the SWI/SNF-related ACRs, which are grouped into four conserved families of INO80/SWR1, SWI/SNF, CHD, and ISWI, have been proven to mediate chromatin remodeling for DDR signaling and DNA repair in *Arabidopsis thaliana* [37,43].

INO80/SWR1: The yeast INO80 complexes containing ACTIN-RELATED PROTEIN5 (ARP5) and ARP8 are recruited to DSB sites by γH2AX, phosphorylated by MEC1/TEL1 kinases, ATM/ATR in mammals, and then facilitate DSB repair by interacting with the histone core and modulating the chromatin structure [84,85,86]. The *Arabidopsis* INO80 (AtINO80) plays a dual role in the transcription and HR repair of DNA damage [87]. The AtINO80-mediated chromatin-remodeling is therefore crucial in genome stability maintenance and in plant development [88]. The ARP5, a conserved subunit of the INO80 chromatin-remodeling complex in yeasts, mammals, and plants, is responsible for the multicellular development and DNA repair in *Arabidopsis thaliana* [89]. In contrast, the yeast SWR1 complexes are closely related with the INO80 but have distinctive roles in DSB repair and checkpoint activation [90]. The *Arabidopsis* SWR1 (AtSWR1) complexes containing core subunits, PHOTOPERIOD-INDEPENDENT EARLY FLOWERING1 (PIE1), ARP6, and SWR1 COMPLEX SUBUNIT6 (SWC6), are involved in the substitution of H2A by H2AZ in nucleosomes [91,92]. The AtSWR1 complex is important for somatic HR repair and meiosis [93]. In addition, the AtSWR1 subunits and H2AZ may have non-redundant functions in plant immunity and gene regulation in *Arabidopsis* [94].

SWI/SNF: The *Arabidopsis* SWI/SNF chromatin remodeler BRAHMA (AtBRM) complex is known to function in DDR and HR repair [61]. However, the specific roles of the AtBRM and its putative subunits SWI3, CHC1, ARP4, and BSH in DDR signaling and DNA repair remain to be experimentally characterized [21,37].

CHD: *Arabidopsis* encodes four CHD family chromatin remodelers: A CHD1 homolog CHROMATIN REMODELING5 (CHR5) and three CHD3 homologs PICKLE (PKL), PKR, and PKR2 [37,95]. These CHD proteins regulate plant development and the stress response by controlling gene expression. Recently, additional roles of the *Arabidopsis* CHR5 are being disclosed in remodeling nucleosome occupancy and regulating plant immune response [96]. However, there is no evidence showing whether the CHD proteins play a role in plant DDR [21].

ISWI: The *Arabidopsis* CHR11 and CHR17 proteins represent plant ISWI chromatin remodelers [21,37]. The AtISWI proteins are important in the formation of the nucleosome distribution patterns, which are associated with gene expression [97]. However, a putative role of the ISWI chromatin remodelers in plant DDR still remains unidentified [21].

Uncategorized: The *Arabidopsis* RAD54 (AtRAD54), which belongs to the SWI2/SNF2 family of chromatin remodelers, plays an important role in DDR and HR repair by modulating the chromatin structure and interacting with AtRAD51 [44,61]. Another SWI2/SNF2 family chromatin remodeler DDM1 contributes to the methylation and stable silencing of transposable elements by allowing DNA methyltransferases to access H1-containing heterochromatin [98]. The *Arabidopsis* DDM1 is involved in the homology directed repair such as single-strand annealing (SSA) and HR at DSB sites by modulating the chromatin structure [39]. In contrast, The STRUCTURAL MAINTENANCE OF CHROMOSOMES (SMC) complex proteins MIM/AtRAD18 and AtRAD21.1 are required for the alternative KU-independent NHEJ repair in *Arabidopsis* [99]. In addition, the *Arabidopsis* DEFECTIVE IN RNA-DIRECTED DNA METHYLATION1 (DRD1) and SNF2-RING-HELICASE-LIKE1 (FRG1)/FRG2, paralogs of RAD5/16 in yeasts, which are components of the RNA-directed DNA methylation (RdDM) pathway, may function in plant DDR as chromatin remodelers [37,61,100].

### 5.2. Histone Modifiers

During DNA damage sensing, signaling, and repair, various types of histone codes that facilitate the accessibility of the sensing and repair machinery, are generated by post-translational modifications (PTMs) of histone proteins, such as phosphorylation, methylation, acetylation, and ubiquitylation [136,137]. Histone PTMs affect chromatin structure and dynamics in gene transcription, DNA replication, and repair by modulating histone–DNA and histone–histone interactions or by cooperating with protein effectors having histone-binding domains or histone readers [138,139,140]. As well as chromatin remodelers, histone modifying enzymes and histone chaperones belong to the protein effectors interacting with histone PTMs.

Histone modifications, which are mediated by the H3K9 methyltransferase KRYPTONITE (KYP), H3K4 demethylase JMJ14, and histone acetyltransferase HAC1, correlate with gene expression and signaling in *Arabidopsis* [141,142]. In DDR, expression of some *Arabidopsis* DNA repair genes encoding CIPK11, RPA1E, GMI1, RAD51, and AGO2 are associated with H3K4me3 or H3K9 acetylation (H3K9ac) after γ-irradiation [78]. Histone acetylation promoted by DSBs facilitates opening of chromatin structures, therefore acetylation of histones H3 and H4 is subject to dynamic changes in response to DNA damage induced by γ-irradiation [38,143]. Although evidence for specific roles of histone modifiers, including histone modifying enzymes and histone chaperones has been accumulating in yeast and mammalian DDR and DNA repair, histone PTMs in plant DDR are still poorly understood as described below. A recent profiling of interactions between histone peptides and putative reader domains in *Arabidopsis thaliana* suggests a broad range of histone modifiers to recognize, bind, and modulate histone PTMs in plants [144].

Histone phosphorylation: The conserved and phosphorylated motif of H2AX centering on serine four residues from the carboxyl terminus indicates a conserved function in DDR throughout evolution among animals, plants, and fungi [101]. Phosphorylation of H2AX, which is an evolutionally conserved response to DSBs, is mediated by MEC1/TEL1 in yeasts or ATM/ATR kinases in mammals and plants and is necessary for the recruitment of repair machineries at DSB sites [102,103].

Histone methylation: The yeast SET domain protein SET9 mediates H4K20 methylation, which is required for the localization of the checkpoint protein Crb2 to DNA damage sites in DDR [145]. The yeast methyltransferase SET1 can methylate H3K4 to facilitate the NHEJ repair of DSBs and the genome stability of yeast cells to DNA damaging agents [146]. The human trimethyltransferase SETD2 is responsible for trimethylation of H3K36 (H3K36me3), which is required to recruit the MMR recognition factor MutSα through direct interaction with the PWWP domain of MSH6, a subunit of MutSα [73]. In *Arabidopsis*, the H3K27 methyltransferase CURLY LEAF (CLF), a homolog of the polycomb-group (PcG) protein EZH2 in mammals, is associated with the epigenetic regulation of somatic and meiotic HR repair [104]. In addition, the *Arabidopsis* H3K27 monomethyltransferases ARABIDOPSIS TRITHORAX-RELATED5 (ATXR5) and ATXR6 prevent over-replication-associated heterochromatic DNA damage by maintaining the H3K27me1 probably on histone H3 variant H3.1 [41,105].

Histone acetylation and ubiquitylation: The yeast histone acetyltransferase ESA1 and deacetylases (HDACs) RPD3 and HOS2 are required for the transcriptional regulation of DNA damage-inducible genes *RNR3* and *HUG1* by modifying promoter-interacting nucleosomes [147]. In human cells, the histone acetyltransferase TIP60 and ubiquitin-conjugating enzyme UBC13 complex mediates the release of H2AX from damaged chromatin by acetylating and then ubiquitylating H2AX [10]. The human histone acetyltransferase MOF has a critical role in DDR and HR/NHEJ-mediated DSB repair by catalyzing H4K16 acetylation (H4K16ac) [148]. The human TIP60 is recruited to chromatin through interaction with DNA damage-induced H3K36me3 and its reader protein LEDGF and is involved in transcriptional regulation and DDR signaling by mediating H4K16ac [149]. In *Arabidopsis*, the histone acetyltransferases HAM1 (a homolog to the human TIP60) and HAG3, which belong to the MYST or GNAT family, respectively, participate in UV-B-induced DDR signaling and DNA repair by negatively regulating the expression of DNA repair enzymes [106,107]. In contrast, the *Arabidopsis* histone acetyltransferases HAC1 and HAF1, which belong to the p300/CBP or TAFII250 family, respectively, have crucial roles in UV-B signaling rather than in DNA repair [108]. In addition, the *Arabidopsis* HDACs including HDA2, HDA6, and HDA19 regulate gene expression in abiotic stress responses by modulating H3K9K14ac or H3K9ac [109], therefore may be associated with DDR signaling and/or DNA repair similarly to the yeast HDACs.

Histone chaperone: The evolutionally conserved histone chaperones are divided into two types, H2A–H2B and H3–H4 [113]. Nucleosome assembly protein1 (NAP1) and FACT chaperones belong to the H2A–H2B type, while CAF-1, ANTI-SILENCING FUNCTION1 (ASF1), and HISTONE REGULATORY HOMOLOG A (HIRA) are the H3–H4 chaperones. The *Arabidopsis* H2A–H2B type chaperones NAP1, NAP1-RELATED PROTEIN (NRP1), and NRP2 are involved in nucleosome disassembly/reassembly for somatic HR [110]. The activity of NRP1 as a histone chaperone is inhibited by cytochrome c and core histones competing for its histone-binding domains during DDR [111]. The histone chaperone FACT functions as a key protein in chromatin remodeling for the initiation of HR repair in human cells by facilitating ubiquitylation of histone H2B through interaction with the E3 ubiquitin ligase RNF20, while it is required for targeting of DEMETER (DME) DNA glycosylase to heterochromatin during reproduction in *Arabidopsis* [47,112]. In contrast, the H3H4 histone chaperone CAF-1 contributes to heterochromatin formation, mitotic chromosome integrity, and transcriptional regulation of HR/NHEJ repair genes in *Arabidopsis* by facilitating the incorporation of histones H3 and H4 onto newly synthesized DNA [114,115]. BRU1, a linker between DDR and epigenetic gene silencing, may cooperate with the CAF-1 in the replication and stabilization of chromatin structure [116]. The ASF1 chaperone exists in *Arabidopsis* as two homologues AtASF1A and AtASF1B [113]. Both AtASF1A and AtASF1B proteins bind histone H3 and play crucial but redundant roles in chromatin replication, maintenance of genome integrity, and cell proliferation. The H3–H4 chaperone HIRA that deposits histone H3.3 into chromatin is required for transcriptional reactivation in damaged chromatin regions after UV-C damage in human cells and is involved in transcriptional dynamics during asexual reproduction and environmental stress response in *Arabidopsis* [117,118].

### 5.3. DNA (De-)Methylation Enzymes

Histone modifications associate with DNA methylation in *Arabidopsis* [141,142,150]. *Arabidopsis* has developed a multi-layered DNA methylation/demethylation system that contributes to transcriptional silencing, imprinting, and genome stability [119]. In contrast to mammalian genomes, in which only CG sites are methylated by the maintenance DNA METHYLTRANSFERASE1 (DNMT1) and de novo DNMT3A/B methyltransferases, *Arabidopsis* genomes contain three types of DNA methyltransferases METHYLTRANSFERASE1 (MET1), CMT3, and DOMAINS REARRANGED METHYLTRANSFERASE2 (DRM2) or CMT2 to methylate CG, CHG, and CHH sites, respectively [98,119]. In addition, there are four DNA glycosylases or demethylases in *Arabidopsis* including DME, REPRESSOR OF SILENCING1 (ROS1), DEMETER-LIKE2 (DML2), and DEMETER-LIKE3 (DML3) [120]. The cytosine methylation is highly conserved at CG sites in genes and CHH sites in repeat regions [121]. The loss of DNA methylation may influence the evolution of plant genomes by altering the recombination landscape through the control of meiotic HR [122].

DNA methylation/demethylation: DNA methylation in eukaryotes marks and silences the recombinant genes induced by HR repair [151]. In other words, HR repair modifies DNA methylation of the repaired segments and alters the local histone H3 methylation as well as chromatin structure, allowing permanent variation of gene expression in somatic cells [152]. DNMT1 is recruited to DNA repair sites via a PROLIFERATING CELL NUCLEAR ANTIGEN (PCNA) and inhibits the expression of the repaired genes by methylating CG sites [151,153]. Therefore, the increased expression of *DNMT1* correlates with the decreased expression of a DNA repair gene, *MLH1*, in human bladder cancer [154]. DNMT1-deficient cells have profound defects in DDR and DSB formation that are induced by a cytidine analog and DNA methylation inhibitor 5-aza-2′-deoxycytidine, while DNMT3B-deficient cells show mild effects [155]. The cytidine analogs zebularine and 5-aza-2′-deoxycytidine generate DNA-protein crosslinks by covalently trapping DNMTs independent of DNA methylation changes, and the repair requires ATM/ATR kinases and a SMC5/6 complex to activate HR [156]. Since DNMT inhibitors induce DNA damage and radiosensitize human cancer cells [157], DNMTs are responsible for the delayed genome instability and radioresistance in stem cell-like cancer cells [158]. Moreover, the age-related hyper-methylation of gene promoters in intestinal stem cells is also attributed to proliferation-associated DNA damage and repair [159]. There is a lack of evidence in plants showing a correlation between DNA (de)-methylation enzymes and DDR/DNA repair. The *Arabidopsis* ROS1 influences DDR to genotoxic stress as a repressor of transcriptional gene silencing (TGS) by demethylating the target promoters [124]. In contrast, the global non-CG hypomethylation in the *Arabidopsis* genome after gamma irradiation, which is attributed to the transcriptional suppression of the *CMT3* gene, is not causally associated with either DDR or DNA repair [77]. Meanwhile, both the CG and non-CG methylation are increased about 10% in the Chernobyl radio-contaminated soybean seedlings versus the control [123]. As reported recently, overexpression of genes encoding DNA (de)-methylation enzymes such as *MET1* may help to identify their putative roles in DDR and DNA repair [160].

DNA methylation regulators: The *Arabidopsis* RPA2 protein with conserved DNA replication and repair motifs, is involved in TGS but is dispensable for small RNA accumulation and DNA methylation [125]. In contrast, the XERODERMA PIGMENTOSUM C (XPC) DNA repair complex coordinates global and locus-specific DNA demethylation along with active transcription during somatic cell reprogramming by cooperating with THYMINE DNA GLYCOSYLASE (TDG), a BER enzyme for removal of all known derivatives of 5-methylcytosine (5-meC) [161]. The *Arabidopsis* DNA repair protein X-RAY CROSS-COMPLEMENTING1 (XRCC1) functions in active DNA demethylation by interacting with ROS1 and a DNA 3′-phosphatase ZDP as a BER component to facilitate 5-meC excision, gap tailoring, and DNA ligation [128]. Another DNA repair factor, DNA DAMAGE BINDING PROTEIN2 (DDB2), is involved in active DNA demethylation and DNA methylation maintenance as a transcriptional regulator of *ROS1* and *DML3* and influences de novo DNA methylation by forming functional DDB2-AGO4-small interfering RNA (siRNA) complexes [129]. The DDB2 also inhibits 5-meC glycosylase activity of ROS1 and stimulates post-incision events in the DNA demethylation pathway by interacting with the ZDP and the 3′-phosphodiesterase APE1L [130]. In addition, the METHYLENETETRAHYDROFOLATE DEHYDROGENASE1 (MTHFD1) plays an important role in TGS by controlling non-CG DNA methylation and repressive histone H3K9 methylation in *Arabidopsis* [126]. The *Arabidopsis* histone H3 variant H3.3 regulates gene body DNA methylation associated with transcriptional activity by preventing recruitment of linker histone H1 for chromatin folding [127]. In contrast, the *Arabidopsis* FACT complex is required for DME-mediated DNA demethylation at DME-target loci in heterochromatic regions enriched with H3K9me2 and H3K27me1 [112].

### 5.4. Noncoding RNAs

Noncoding RNAs (ncRNAs), which include miRNAs, siRNAs, DSB-induced RNAs (diRNAs), DNA damage response RNAs (DDRNAs), piwi-interacting RNAs (piRNAs), and long ncRNAs (lncRNAs), are emerging new players in DDR and DNA repair [162,163,164]. The putative roles of ncRNAs in the regulation of HR and NHEJ repair of DSBs are recently beginning to be defined [165]. miRNAs, siRNAs, diRNAs, DDRNAs, and piRNAs, which are small ncRNAs (sncRNAs) of about 20–30 nt, are involved in DDR and genome stability via TGS, post-TGS (PTGS), and chromatin regulation [13,165]. Multiple functions of lncRNAs with >200 nt are also associated with DDR and an oxidative stress response in human cancer progression [165,166]. Transposable elements (TEs) are the major constituents of eukaryotic genomes, especially occupying more than 80% in some plant genomes, and therefore both sncRNAs and lncRNAs are derived from TEs in the plant stress response [167]. Although the database of plant ncRNAs has been greatly updated [168], the roles of major ncRNAs—miRNAs, siRNA, diRNAs (or DDRNAs), and lncRNAs except for piRNAs in animals—in plant DDR and DNA repair, remain poorly identified as described below.

miRNAs: miRNAs are highly conserved sncRNAs and regulate protein expression and multiple intracellular processes in human cells as a cellular defense mechanism against genotoxic oxidative stress [169]. DNA damage modulates miRNA expression at the transcription and post-transcription levels as well as miRNA degradation, while miRNAs regulate DDR sensors, transducers, and effectors [163]. In particular, miR-24/138, miR-182, miR-101/421, and miR-125b/504 are key regulators to target γH2AX, BRCA1, ATM, or P53, respectively, in IR-induced DDR [79,170]. In addition, miR-96/155/506 and miR-124/526/622b are involved in HR or NHEJ repair by targeting RAD51 or KU70/80, respectively [165,171,172]. miRNAs are also important regulators of gene expression in plant stress responses as well as plant growth, development, and maintenance of genome integrity [131]. For example, the *Arabidopsis* miR156/159/160/166/390/393/398 are UV-B- or oxidative stress-responsive and participate in the regulatory network of plant stress responses [173,174]. However, there is no direct evidence supporting a correlation between miRNAs and DDR/DNA repair in plants. Plant specific and genotoxic stress-responsive miRNAs including the IR-induced *Arabidopsis* miR840 and miR850 remain to be further characterized in terms of their functions in DDR and DNA repair [80].

siRNAs and diRNAs: siRNAs are produced from long double-stranded RNA (dsRNA) or RNA-DEPENDENT RNA POLYMERASE (RDR)-synthesized dsRNA through cleavage by the endonuclease DICER and then loaded onto an AGO protein to mediate TGS and PTGS [13]. The siRNAs of Alu interspersed repetitive elements stabilize the genome and prevent endogenous DNA damage by increasing Alu element methylation in human cells [175]. In *Arabidopsis*, the 24-nt siRNAs, which are synthesized and processed by RNA POLYMERASE IV (POL IV), RDR2, and DICER-LIKE3 (DCL3), form a complex with DDB2 and AGO4 to regulate de novo DNA methylation in RdDM pathway [132]. In addition, the UV-induced 21-nt siRNAs, which are produced by concerted action of POL IV, RDR2 and DCL4, are required for the recognition and repair of DNA photoproducts by forming a chromatin-bound complex with DDB2 and AGO1 [132]. The 21-nt diRNAs or DDRNAs are also produced in *Arabidopsis* and mammals by DNA damage and are required for DSB repair or DDR activation [133,134]. diRNAs are recruited at DSB sites via interaction with AGO2 to repair lesions in *Arabidopsis* [133], while DDRNAs are processed by DROSHA and DICER to facilitate DDR foci formation for DDR activation in human, mouse, and zebrafish [176]. sncRNAs require specific AGO proteins as key players in their production and function. Three phylogenetic clades of *Arabidopsis* AGO proteins—AGO1/AGO5/AGO10, AGO2/AGO3/AGO7, and AGO4/AGO6/AGO8/AGO9—play distinct roles in siRNA, miRNA, and/or RdDM pathways [177]. In particular, AGO1, AGO2, AGO4, AGO5, and AGO9 are associated with DNA repair and genome stability as well as the biogenesis and function of sncRNAs [177,178].

lncRNAs: lncRNAs originate from thousands of loci across animal and plant genomes and are generally classified into three groups: (i) Long intergenic ncRNAs (lincRNAs), (ii) intronic ncRNAs (incRNAs), and (iii) natural antisense transcripts (NATs) [179,180]. Many of them are produced by POL II and the plant-specific POL IV and V. lncRNAs form ribonucleoprotein (RNP) complexes with chromatin regulators and target the RNP complexes to appropriate locations in the genome by functioning as decoys, scaffolds, guides and *cis*-/*trans*-acting enhancers [181]. The extensive networks of lncRNAs interacting with numerous chromatin components and regulators play multiple roles in gene expression control, scaffold formation, and epigenetic control [14,182]. Therefore, lncRNAs influence gene expression via chromatin modification as well as transcriptional and post-transcriptional regulation [163]. In DDR, one of the X-ray-induced lincRNAs in human cells mediates DDR signaling by regulating DDR gene expression in a P53-dependent manner [183]. The X-ray-induced lncRNAs are synthesized by POL II binding to the MRN complex at DSBs, and control DDR activation and DNA repair by facilitating DDR foci formation through interaction with DDRNAs at individual DSBs [184]. In addition, the human lncRNAs—DDSR1, lncRNA-JADE, TERRA, LINP1, WRAP53α, and linc-ROR—participate in DDR by targeting key components of HR/NHEJ repair, such as BRACA1, MRE11, KU70/80, and P53 [185]. lncRNAs are poorly conserved in animals and plants, and they are highly tissue specific and responsive to biotic and/or abiotic stresses in plants [180]. Therefore, there is a lack of evidence showing the putative roles of lncRNAs in plant DDR. The *Arabidopsis* lncRNA TER2 is involved in maintaining genome integrity by inhibiting TERT, the catalytic subunit of telomerase, in concert with the canonical TER1 under DSB-inducing genotoxic stress [135]. However, only less than 0.6% of TEs and lncRNAs in *Arabidopsis* respond to X-ray induced DNA damage, and most of them (≥95%) are regulated in an ATM-dependent manner by the ATM-downstream factors, including BRCA1, DRM1, JMJ30, and AGO2 [186].

## 6. Concluding Remarks and Perspectives

Epigenetic regulation of DDR, including chromatin remodeling, DNA methylation, histone modification, and RNA silencing, has been extensively explored in yeasts and mammalian cells, and several epigenetic modifiers with similar or novel functions have recently been identified to play a role in plant DDR. Since plants have a relatively huge genome size and face different kinds of unavoidable and extreme environmental stresses during their life cycle, they may need highly sophisticated epigenetic regulation mechanisms to overcome such threats and to maintain genome stability. In fact, the putative roles of the recently identified epigenetic modifiers in plants imply that different types of genetic and epigenetic regulation machineries concertedly cooperate during DNA damage recognition and repair in DDR. Our knowledge of plant DDR has recently improved owing to elucidation of the putative roles of some key players such as SOG1, RBR1/E2FA, and NAC103 in the transcriptional regulation of DDR genes. Although I have reviewed the relevant articles, I have speculated on the epigenetic regulation of DDR and DNA repair in plants, as data are scarce on epigenetic modifiers that contribute to plant DDR and DNA repair. Therefore, novel or known plant epigenetic modifiers need to be further explored in terms of epigenetic regulation of DDR and DNA repair via chromatin remodeling, histone modification, DNA methylation/demethylation, and/or ncRNA-mediated silencing.

## Figures and Tables

**Table 1:** DNA repair mechanisms associated with chromatin remodeling in eukaryotic cells. 1. *Arabidopsis*, 2. human or mammalian, and 3. yeast.

DNA Repair	Chromatin Modifier	Action Mechanisms	Reference
HR	1–3. RAD54, 1. DDM1	Induce chromatin remodeling through interaction with RAD51	[39,43,44]
	1. MMS21	Function as a critical subunit of the SMC5/6 complex	[45,46]
	2. RNF20	Ubiquitylate H2B through interaction with FACT	[47]
NHEJ	1–3. INO80	Involved in nucleosome disassembly around DSBs	[21,43,48]
	2. ASF1A, HIRA, CAF-1	Involved in nucleosome reassembly	[48]
	3. RSC	Facilitate NHEJ through interaction with MRE11 and KU70/80	[49]
NER	1–3. ACRs	Reorganize chromatin structure and control DNA accessibility	[43,50]
	2–3. SWI, INO80, ARP5	Promote the removal of UV lesions through interaction with RAD4/23	[51,52,53]
BER	1–3. ACRs	Affect accessibility of BER enzymes to nucleosomal DNA	[54,55,56]
	3. SWI/SNF	Induce chromatin remodeling to facilitate the removal of oxidative 8-oxoG lesions	[57,58]
MMR	1–3. Chromatin and histone modifiers	Affect nucleosome assembly and disassembly	[59]
	2. PCNA	Involved in chromatin assembly through interaction with MSH6 and CAF-1	[60]

**Table 2:** Epigenetic modifiers in plant DDR and DNA repair. This summarizes the representative examples for each epigenetic modifier in correlation with DDR or DNA repair in plants. ‘Unknown in DDR’ means that there is no evidence to correlate the epigenetic modifier with DDR in plants, although it may exist in yeasts or animals.

Epigenetic Modifier	Member or Subunit	Functions	Reference
*Chromatin remodeler*			
INO80/SWR1	INO80, ARP5, ARP8	Involved in HR repair of DNA damage, maintenance of genome stability, and somatic HR and meiosis	[87,88,89,93]
	SWR1, PIE1, ARP6, SWC6	Involved in substitution of nucleosomal H2A by H2AZ and gene regulation	[91,92,94]
SWI/SNF	BRM, SWI3, CHC1, ARP4, BSH	Function in DDR and HR repair through unknown mechanisms	[21,37,61]
CHD	CHR5, PKL, PKR, PKR2	Unknown in DDR, but involved in nucleosome remodeling and gene regulation	[21,37,95,96]
ISWI	ISWI, CHR11, CHR17	Unknown in DDR, but involved in nucleosome distribution	[21,37,97]
Uncategorized	RAD54	Involved in DDR and HR repair by modulating chromatin structure through interaction with RAD51	[44,61]
	DDM1	Involved in DDR and HR repair as well as in methylation and silencing of transposable elements	[39,98]
	MIM/RAD18, RAD21.1	Involved in KU-independent NHEJ repair by constituting SMC complex	[99]
	DRD1, FRG1/FRG2	Unknown in DDR, but involved in chromatin remodeling as RdDM components	[37,61,100]
*Histone modifier*			
Kinase	ATM, ATR	Facilitate recruitment of repair machineries at DSB sites by phosphorylating H2AX and start DDR	[101,102,103]
Methyltransferase	CLF, ATXR5, ATXR6	Involved in regulation of somatic and meiotic HR repair or in preventing overreplication-associated heterochromatic DNA damage as a H3K27 methyltransferase	[41,104,105]
Acetyltransferase/deacetylase	HAM1, HAG3, HAC1, HAF1	Involved in UV-B-induced DDR signaling and/or DNA repair	[106,107,108]
	HDA2, ADA6, ADA19	Unknown in DDR, but involved in gene regulation in abiotic stress responses as a H3K9 deacetylase	[109]
Chaperone	NAP1, NRP1, NRP2, FACT	Involved in nucleosome remodeling for somatic HR or in targeting of DME as a H2A-H2B chaperone	[47,110,111,112]
	CAF-1, ASF1, HIRA	Contribute to genome integrity/stability and transcriptional regulation of HR/NHEJ genes as a H3-H4 chaperone	[113,114,115,116,117,118]
*DNA (de-)methylation enzyme*			
Methyltransferase	MET1, CMT2, CMT3, DRM2	Unknown in DDR, but correlate with meiotic recombination landscape, global non-CG hypomethylation after γ-irradiation, or increased CG/non-CG methylation in Chernobyl soybean seedlings	[77,98,119,120,121,122,123]
Demethylase	ROS1, DME, DML2, DML3	ROS influences DDR to genotoxic stress as a TGS repressor	[120,124]
Regulator	RPA2, MTHFD1, H3.3	Unknown in DDR, but involved in DNA methylation for TGS or transcriptional regulation	[125,126,127]
	XRCC1, FACT	Unknown in DDR, but involved in active DNA demethylation by interacting with ROS1 or DME	[112,128]
	DDB2	Involved in DNA methylation by interacting with AGO4-siRNA or active DNA demethylation by regulating ROS1	[129,130]
*ncRNA*			
miRNA	miR156/159/160/166/390/393, miR398, miR840, miR850	Unknown in DDR, but involved in stress responses as well as development and maintenance of genome integrity	[80,131]
siRNA	24-nt siRNA, 21-nt siRNA	Involved in de novo DNA methylation with DDB2-AGO4 or repair of DNA photoproducts with DDB2-AGO1	[132]
diRNA	21-nt diRNA	Involved in DSB repair or DDR activation via interaction with AGO2	[133,134]
lncRNA	TER2	Involved in maintenance of genome integrity by inhibiting TERT under genotoxic stress	[135]

## Abbreviations

ACRs	ATP-dependent chromatin remodelers
AGO	Argonaute
ATM/ATR	Ataxia telangiectasia mutated /ATM and rad3-related
BER or NER	Base or nucleotide excision repair
CHD	Chromodomain helicase DNA
DDR	DNA damage response
diRNAs or DDRNAs	DSB-induced RNAs or DNA damage response RNAs
DME/DML2/DML3	Demeter/Demeter-like2/Demeter-like3
DSBs	DNA double-strand breaks
HDACs	Histone deacetylases
HR	Homologous recombination
INO80	Inositol-requiring mutant80
ISWI	Imitation switch
MET1/CMT2/3/DRM2	Methyltransferase1/Chromomethylase2/3/Domains rearranged methyltransferase2
miRNAs	MicroRNAs
MMR	Mismatch repair
NHEJ	Non-homologous end-joining
PTMs	Post-translational modifications
RAD54	Radiation-sensitive54
RBR1	Retinoblastoma related1
RdDM	RNA-directed DNA methylation
ROS1	Repressor of silencing1
siRNAs	Small interfering RNAs
sncRNAs or lncRNA	Small or long noncoding RNAs
SOG1	Suppressor of gamma response1
SWI/SNF	Switch/Sucrose nonfermentable
TGS	Transcriptional gene silencing
